# Kawasaki Related Coronary Artery Disease Refractory to Angioplasty: The Role of Intravascular Shockwave Lithotripsy

**DOI:** 10.7759/cureus.19020

**Published:** 2021-10-25

**Authors:** Teodora Donisan, Amy Mertens, Sayed Luay

**Affiliations:** 1 Department of Internal Medicine, Beaumont Hospital, Royal Oak, Royal Oak, USA; 2 Department of Cardiology, Beaumont Hospital, Royal Oak, Royal Oak, USA; 3 Department of Cardiology, Beaumont Hospital, Troy, Troy, USA

**Keywords:** severe calcified lesion, coronary vasculitis, angioplasty, coronary aneurysm, intravascular lithotripsy

## Abstract

Kawasaki disease is a systemic vasculitis with frequent coronary artery involvement, associated with coronary artery aneurysms (CAAs) even if appropriately treated. Patients with CAA have a high risk for cardiovascular complications and frequently undergo repeated coronary interventions. Coronary lesions associated with Kawasaki can be heavily calcified, presenting a therapeutic challenge. We discuss the case of a 27-year-old patient who developed CAA and severe coronary artery calcifications despite appropriate treatment after Kawasaki disease when he was two years old. The coronary stenosis was heavily calcified and failed treatment with cutting balloons, orbital atherectomy, and rotational atherectomy, but yielded after being treated with intravascular lithotripsy. The patient was treated with drug-eluting stent and covered stent to exclude the CAA, with a good final result.

## Introduction

Kawasaki disease is a systemic vasculitis affecting children that can be complicated by coronary artery aneurysms (CAAs) in 5% of patients despite adequate anti-inflammatory treatment [1]. The natural history of CAA in Kawasaki disease is heterogeneous. CAA can remodel, remain unchanged, and progress to stenotic lesions or thrombosis, leading to increased cardiovascular morbidity. Intracoronary imaging studies demonstrated endothelial fibrosis and cellular infiltration, but the vascular destruction is worse in vascular segments with persistent or regressed CAA: media layer destruction, calcification, neovascularization, and white thrombi [2]. There is also evidence of persistent arterial wall inflammation decades after the acute disease [3]. Risk factors for CAA include Asian and Hispanic ethnicities and male gender. CAA most frequently occurs in the right coronary artery (RCA) in >40% of cases, followed by the left anterior descending artery (LAD) and the left circumflex artery (LCx) [4]. Patients with CAA can be asymptomatic, but they can present with angina, acute coronary syndrome, or sudden cardiac death. Acute coronary syndromes after Kawasaki disease occur because of CAA thrombosis or due to progressive stenosis, leading to lesions that are usually heavily calcified and very difficult to treat.

## Case presentation

**Figure 1:**
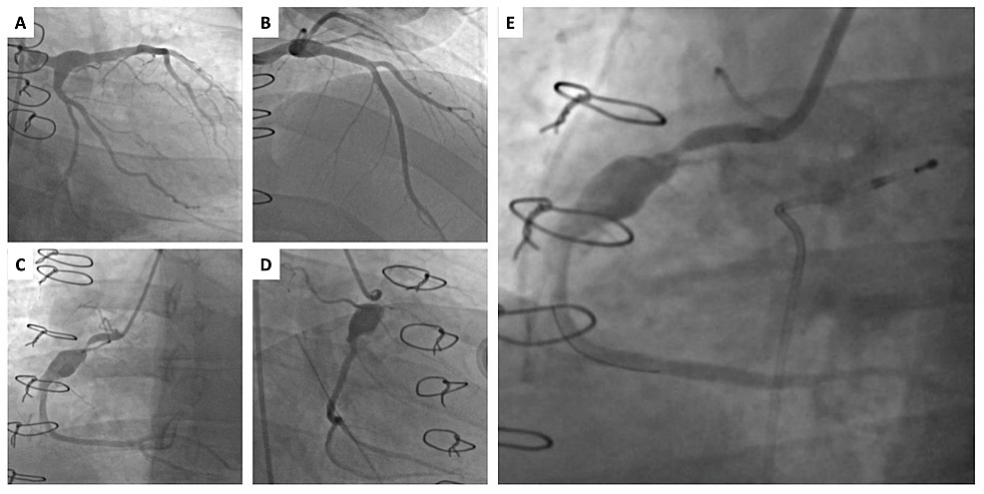
Coronary angiogram before and after attempted angioplasty with cutting balloon and atherectomy (A-B) Left main coronary artery bifurcating into a normal left circumflex artery and a left anterior descending artery with a proximal 5 mm aneurysm. (C-D) Dominant right coronary artery (RCA) with a focal 90% heavily calcified proximal stenosis, followed by a 9.9 mm aneurysm. Distal right internal mammary artery bypass graft small, nonfunctional. (E) Severe residual proximal RCA stenosis, after percutaneous angioplasty with cutting balloon, orbital atherectomy, and rotational atherectomy.

This is a 27-year-old male Hispanic patient with a history of Kawasaki disease at age two (at that time treated with high-dose aspirin and intravenous immune globulin (IVIG)) who presented for worsening progressive dyspnea and fatigue. He underwent coronary angiography at 17-years-old, when he was diagnosed with aneurysms of the RCA and the LAD, and found with a severe calcified stenosis in the proximal RCA. Percutaneous coronary intervention (PCI) was attempted, but the lesion could not be predilated with a balloon, even after inflations of up to 30 atmospheres (atm). He subsequently underwent single-vessel coronary artery bypass grafting (CABG) with right internal mammary artery (RIMA) to the RCA. On the current presentation, he underwent repeat coronary angiography and was found to have graft closure secondary to occlusion of the RIMA, a severe proximal RCA stenosis, and a large poststenotic aneurysm measuring 9.9 mm. The LAD aneurysm was redemonstrated, measuring 5 mm (Figure 1A-1D). Additional workup revealed a normal left ventricular function and a bicuspid aortic valve with mild aortic regurgitation.

A plan was made for proximal RCA rotational atherectomy, followed by covered stent placement, due to the large poststenotic aneurysm. Intravascular ultrasound (IVUS) was initially attempted, but the catheter would not cross the heavily calcified RCA stenosis. Mechanical rotational atherectomy (MRA) was performed with a 1.5 burr, followed by balloon angioplasty, but the lesion would not yield. MRA was performed again with 2.0 burr, followed by orbital atherectomy and cutting balloon angioplasty using wolverine balloon and chocolate balloon, which were all unsuccessful at dilating the lesion. IVUS was performed, revealing a focal, 360 degree heavily calcified stenosis with a lumen of 2 x 2 mm just proximal to the large RCA aneurysm. Multiple balloon angioplasties were attempted again, which were unsuccessful (Figure 1E), so the procedure was aborted.

**Figure 2:**
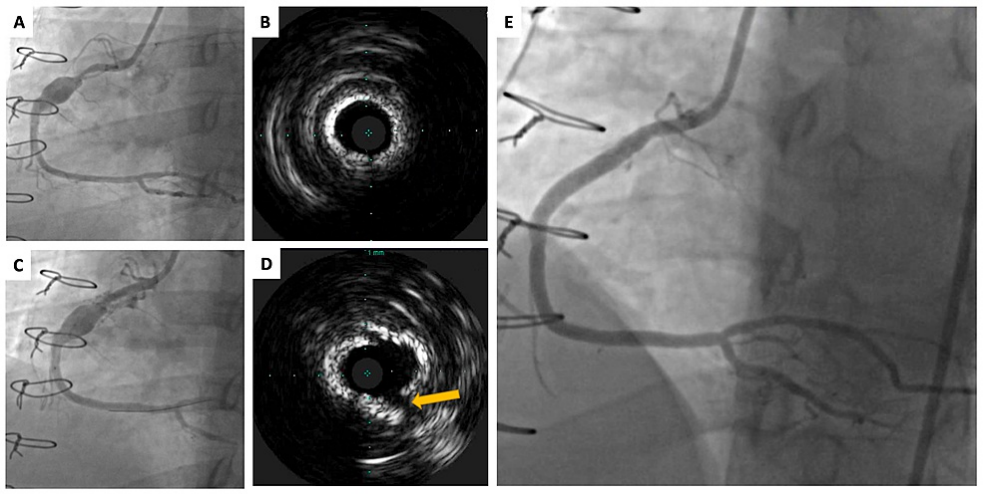
Right coronary artery angiogram and intravascular ultrasound pre- and postangioplasty with intravascular shockwave lithotripsy, drug-eluting stenting, and placement of a covered stent (A) Right coronary artery (RCA) with 90% focal stenosis proximal to the coronary artery aneurysm, three months after the previously shown procedure. (B) Intravascular ultrasound (IVUS) of the proximal RCA stenotic segment showing a severely calcified circumferential lesion. (C) Proximal RCA lesion yielding after intravascular shockwave lithotripsy. (D) IVUS image showing circumferential calcium with a tear in the plaque (yellow arrow). (E) Final RCA result after coronary aneurysm exclusion with a drug-eluting stent scaffold with an overlapping covered stent.

The patient’s dyspnea and fatigue gradually progressed, occurring with minimal exertion. The patient was brought back for a second attempt to treat his RCA stenosis with Shockwave lithotripsy. The proximal RCA stenotic lesion (Figure 2A-2B) was passed nine times using a 3.5 x 40 mm Shockwave lithotripsy balloon, after which postdilation was performed with a 3.5 x 20 mm noncompliant balloon. IVUS revealed circumferential calcium with a tear, suggesting successful treatment of the lesion (Figure 2C-2D). A 4.0 x 38 mm Promus Elite (Boston Scientific, Marlborough, MA, USA) drug-eluting stent was placed across the aneurysm to serve as a bridge, followed by the deployment of two overlapping Papyrus (Biotronik, Berlin, Germany) covered stents (3.5 x 26 mm and 4.0 x 15 mm, respectively). The stents were postdilated with a 4.0 x 8 mm noncompliant balloon, resulting in thrombolysis in myocardial infarction (TIMI) 3 flow (Figure 2E).

## Discussion

Patients with Kawasaki disease and CAA are shown to have a 30-year survival rate of 88%, 59% of them undergo either catheter or surgical interventions at 25 years after the acute disease, and 16% can experience acute coronary syndromes [5]. PCI has a high rate of restenosis or occlusion because of thick intimal hyperplasia [6]. Interventions are also complex because of heavily calcified lesions, requiring high balloon pressures that increase the risk for intimal dissection and new CAA formation [6]. MRA has been used as a treatment option in these cases. CABG has also been used for severe obstructive lesions after Kawasaki disease, with a 20-year patency rate of 87% for internal thoracic artery grafts and 44% for vein grafts [7]. Patients continue to experience frequent cardiac events and require reintervention even after CABG. Furthermore, graft patency may be compromised by competing flow from the native coronary artery [8].

Heavily calcified lesions are often difficult to treat and are associated with significant complications either during PCI (e.g., dissection, perforation) or afterward (e.g., restenosis, stent fractures, stent thrombosis) [9]. Currently available solutions for these types of lesions, such as high-pressure noncompliant balloons, cutting/scoring balloons, and MRA, rely on tissue compression or debulking, in order to fracture calcium and facilitate optimal stent deployment [10]. The overall procedural success rate can be low with lesions containing deep, thick, or eccentric calcifications, and current procedural options (i.e., specialty balloons, atherectomy, and high-pressure noncompliant balloons) have similar clinical outcomes [9].

Intravascular lithotripsy (IVL) was developed as a novel modality to disrupt severely calcified plaque. IVL uses sonic waves traveling from a low-pressure balloon to selectively break superficial and deep calcium deposits, with minimal soft tissue impairment, while improving vessel compliance [11]. Unlike prior plaque treatment methods, IVL does not rely on direct vascular injury and the disrupted calcium remains within the vessel, with a lower risk for distal embolization. The Disrupt CAD III study demonstrated that IVL is a safe and useful procedure for preparing calcified lesions prior to stent deployment [12]. There are concerns that IVL may not be suitable in critical stenoses or in tortuous vessels, as balloon rupture and vessel dissection have been described [13]. Intracoronary imaging with optical coherence tomography or IVUS should be used to pick appropriate lesions for IVL use and to assess need for adjuvant therapy from other plaque modification devices [14].

Although the use of IVL in unyielding lesions, resistant to specialty balloons and rotational atherectomy, has been reported [15], this is the first case discussing the successful use of IVL in patients with CAD caused by an inflammatory vascular disease. Furthermore, there is limited evidence regarding the use of covered stents to treat coronary stenoses proximal and distal to CAA and to prevent thrombus formation within the affected aneurysmal segment, but there have been promising long-term follow-up results [8].

## Conclusions

To the best of our knowledge, this is the first reported use of IVL in severely calcified lesions caused by Kawasaki disease. This case emphasizes the use of modern interventional approaches to address a complex clinical scenario: young patient with a history of Kawasaki disease, multiple coronary artery aneurysms, a severely calcified stenotic RCA refractory to balloon angioplasty, occluded RIMA-RCA bypass graft, and worsening symptoms with declining functional status. IVL was successfully used to treat the calcified lesion, allowing for stenting of the lesion, followed by covered stent deployment to treat the CAA.
